# Supplementary material for: Engineering and Purification of Microcin C7 Variants Resistant to Trypsin and Analysis of Their Biological Activity
Source: Antibiotics (Basel). 2023 Aug 22;12(9):1346. doi: 10.3390/antibiotics12091346 (PMC10525924; doi:10.3390/antibiotics12091346)
Supplement: Supplementary file 1 [file antibiotics-12-01346-s001.zip › antibiotics-2524384-supplementary.pdf]

Supplementary Materials

1. Supplementary Figures

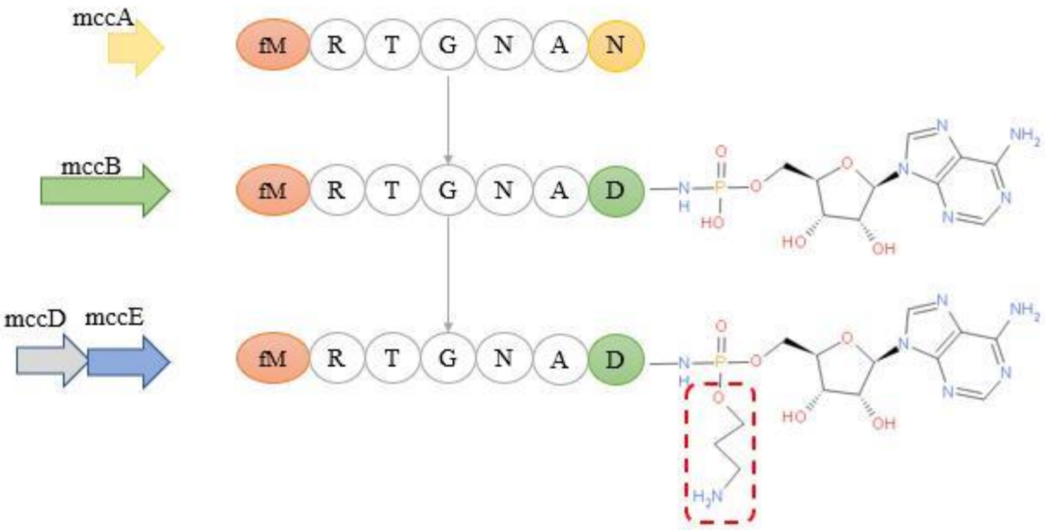

Supplementary Figure S1. Formation process of the McC structure. MRTGNAN is encoded by the *mccA* gene which is the shortest known natural gene. The *mccB* gene is responsible for adenylation of MccA. Aminopropyl is linked to products with MccB-catalyzed adenylation under the joint action of the *mccD* and *mccE*.

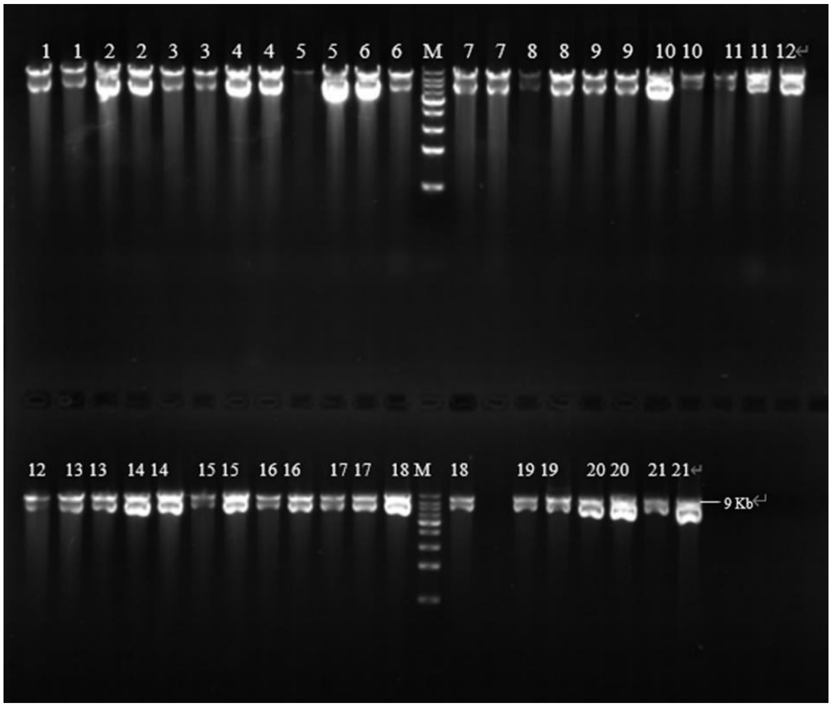

Supplementary Figure S2. The PCR products of McC mutants were transferred into *E. coli* MC4100 competent cells, and the extracted plasmid was subjected to 0.8% agarose gel electrophoresis, and a 9-kb fragment of the same size as expected appeared in each lane. M: DL 10,000 DNA molecular weight marker; 1: PCR products of *mccA* R2A mutant gene; 2: PCR products of *mccA* R2S mutant gene; 3: PCR products of *mccA* R2H mutant gene; 4: PCR products of *mccA* R2W mutant gene; 5: PCR products of *mccA* R2Y mutant gene; 6: PCR products of *mccA* R2L mutant gene; 7: PCR products of *mccA* R2I mutant gene; 8: PCR products of *mccA* R2V mutant gene; 9: PCR products of *mccA* R2M mutant gene; 10: PCR products of *mccA* R2T mutant gene; 11: PCR products of *mccA* R2G mutant gene; 12: PCR products of *mccA* R2C mutant gene; 13: PCR products of *mccA* R2P mutant gene; 14: PCR products of *mccA* R2F mutant gene; 15: PCR products of *mccA* R2N mutant gene; 16: PCR products of *mccA* R2Q mutant gene; 17: PCR products of *mccA* R2K mutant gene; 18: PCR products of *mccA* R2D mutant gene; 19: PCR products of *mccA* R2E mutant gene; 20: PCR products of *mccA* RPT mutant gene; 21: PCR products of *mccA* T3P mutant gene.

2. Supplementary Tables

Supplementary Table S1. Gradient elution conditions for purification

Time (min)	Flow rate (mL/min)	Mobile phase A (%)	Mobile phase B (%)
0	1.5	90	10
5	1.5	90	10
8	1.5	83	17
20	1.5	83	17
20.01	1.5	0	100

Supplementary Table S2. The amount of the remaining McC and its analogues remaining was determined by RPHPLC after trypsin, pepsin and chymotrypsin treated 3 hours.

Mutant	Trypsin treated remaining (%)	Pepsin treated remaining (%)	Chymotrypsin treated remaining (%)
McC7	0	89.32 ± 1.16	41.72 ± 1.07
R2A	84.29 ± 0.54	92.16 ± 1.04	42.16 ± 0.86
R2S	85.33 ± 0.27	84.33 ± 0.77	41.33 ± 1.27
R2H	82.69 ± 0.82	82.29 ± 0.52	42.69 ± 0.69
R2W	0	91.29 ± 1.26	41.29 ± 1.03
R2Y	55.36 ± 0.72	85.22 ± 0.72	45.36 ± 1.18
R2L	0	79.25 ± 0.62	39.25 ± 0.75
R2I	61.27 ± 0.82	91.72 ± 0.87	41.27 ± 0.55
R2V	59.10 ± 1.41	79.01 ± 1.24	39.10 ± 1.05
R2M	48.75 ± 0.93	88.67 ± 0.93	43.75 ± 0.68
R2T	76.67 ± 0.77	86.76 ± 0.77	46.67 ± 0.66
R2G	55.65 ± 0.79	85.89 ± 0.79	45.65 ± 1.31
R2C	0	82.45 ± 0.98	40.85 ± 1.22
R2P	0	86.94 ± 1.24	42.77 ± 0.76
R2F	72.11 ± 0.94	82.33 ± 0.94	41.15 ± 0.98
R2N	0	88.79 ± 0.88	37.69 ± 1.10
R2Q	87.29 ± 1.23	87.64 ± 1.23	49.61 ± 1.19
R2K	0	87.69 ± 0.68	41.35 ± 0.73
R2D	0	83.87 ± 1.42	42.44 ± 1.07
R2E	0	90.72 ± 0.74	38.39 ± 0.97

RPT	74.22 ± 0.79	84.56 ± 0.79	46.39 ± 0.77
T3P	76.75 ± 1.33	86.78 ± 1.33	39.75 ± 0.81
